# Novel Pharmaceuticals and Therapeutics for Tumor Necrosis Factor-Alpha-Resistant Crohn's Disease: A Narrative Review

**DOI:** 10.7759/cureus.65357

**Published:** 2024-07-25

**Authors:** Blake Smith, Haylie Smith, Matthew Machini

**Affiliations:** 1 Medical School, Nova Southeastern University Dr. Kiran C. Patel College of Osteopathic Medicine, Fort Lauderdale, USA; 2 Medical School, Edward Via College of Osteopathic Medicine, Spartanburg, USA; 3 Foundational Sciences, Nova Southeastern University Dr. Kiran C. Patel College of Osteopathic Medicine, Clearwater, USA

**Keywords:** risankizumab, vedolizumab, upadacitinib, crohn's disease, inflammatory bowel disease

## Abstract

Inflammatory bowel disease (IBD) is a medical condition that causes persistent, relapsing inflammation of the gastrointestinal tract. It is an umbrella term encompassing two different conditions: ulcerative colitis (UC) and Crohn’s disease (CD). The standard treatment for patients with moderate to severe CD is tumor necrosis factor-α (TNF-α) inhibitors; however, a subset of CD patients face challenges in regard to this disease’s treatment. Certain populations of patients with CD may exhibit resistance or develop tolerance to TNF-α inhibitor therapy over time. The recurrent gastrointestinal inflammation associated with CD can severely impact the quality of life and lead to complications for those suffering from this condition. The symptomatic flare-ups these subpopulations continue to experience underscores why such a need for alternative therapies is desperately needed. These alternative therapies not only offer potential benefits for those with TNF-α resistance, but CD may also serve as a superior therapy option for those trying to avoid the adverse effects of CD treatments available today. This review aims to explore and investigate the novel drugs and therapies that are being investigated for the treatment of TNF-α resistant CD, such as upadacitinib, risankizumab, vedolizumab, synbiotics, fecal microbiota transplantation (FMT), and stem cell therapy. Upadacitinib is a Janus kinase inhibitor, Risankizumab is a monoclonal antibody targeting interleukin-23, and Vedolizumab is an integrin receptor antagonist. The latest advancements in CD management have shown encouraging results. Some of these novel drugs and therapies not only offer a potential solution for CD patients exhibiting resistance to TNF-α inhibitors but may also provide a superior alternative for individuals prone to opportunistic infections.

## Introduction and background

**Figure 1:**
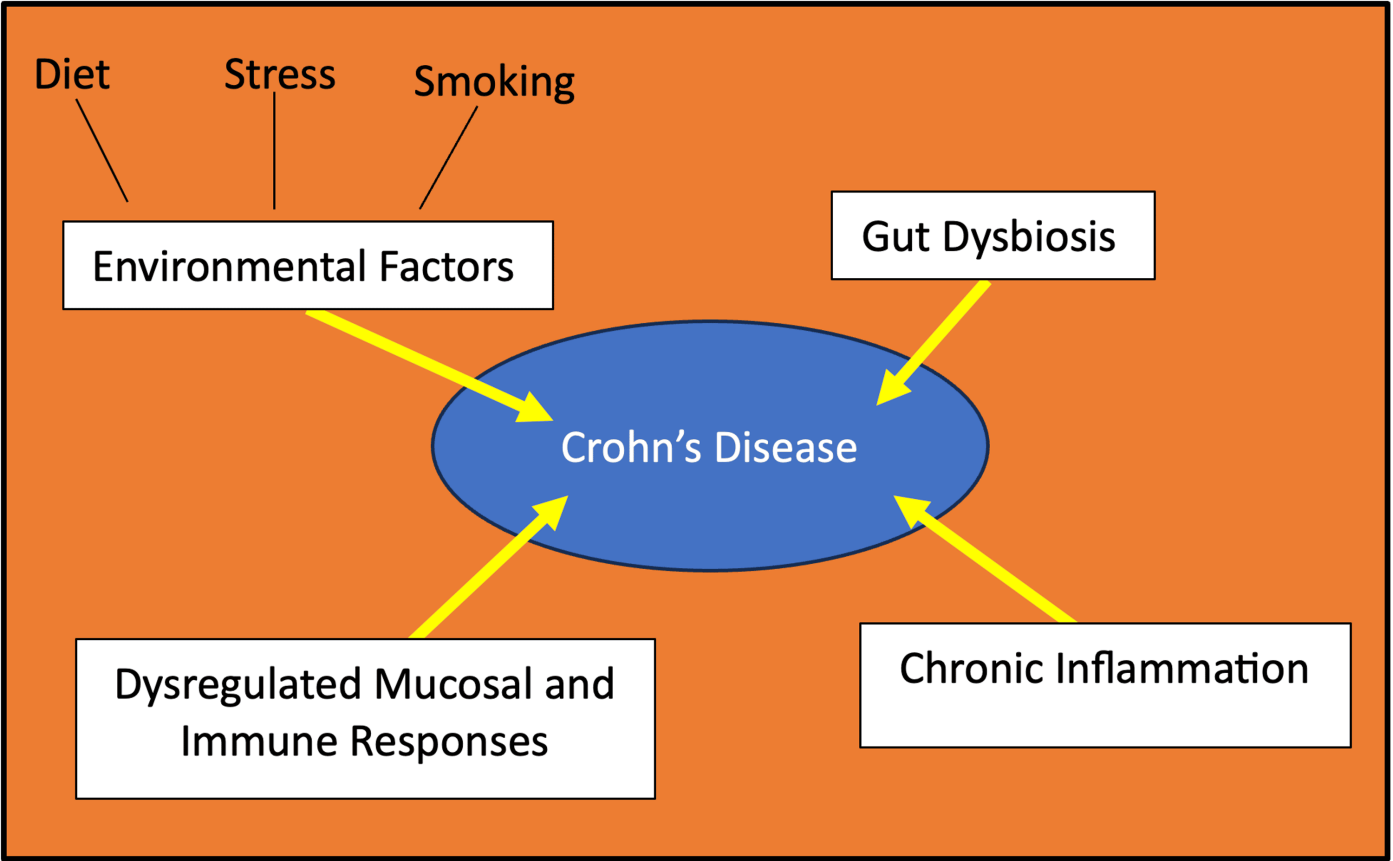
Identifying the various contributors to Crohn's disease Image credits: Blake Smith

Inflammatory bowel disease (IBD) is a broad term designated to describe the chronic, relapsing inflammation of the gastrointestinal tract seen in conditions like Crohn’s disease (CD) and ulcerative colitis (UC). While the exact etiology and pathogenesis of IBD remain elusive, various factors have been implicated in its onset and advancement. Genetic variability, dysregulated host immunity, and environmental influences are all factors that have been proposed to be potential contributors to the development of IBD, as illustrated by Figure 1 [1]. Breaches in the epithelial layer of the intestine with a lack of mucosal healing and regeneration are key characteristics of IBD [1]. Infectious agents, chemical compounds, and diet-mediated dysbiosis can all lead to susceptibility, triggering an inflammatory cascade. A persistent inflammatory cascade may be perpetuated by an immune system that has impaired functions in resolving the inflammatory response to the initial injury. This chronic inflammation can lead to alterations in the tolerance of the commensal microbiota, ultimately leading to tissue damage via autologous signaling [1]. 

Dysregulated innate and adaptive immune responses toward the intestinal microbiota, potentially triggered by an infectious etiology, promote the sustained inflammatory cascade commonly found in IBD. The microbiota plays a role in IBD. Those with IBD had a larger population of Escherichia coli and Bacteroidetes in their normal microbiota [2]. Treatments that increase the quantities of Lactobacillus and Bifidobacterium in the microbiome have been shown to help prevent IBD [2]. Individuals with genetic susceptibilities, such as the nucleotide-binding oligomerization domain-2 (NOD2) variation commonly found in CD, are affected in several ways. The NOD2 gene is typically expressed in the Paneth and other intestinal epithelial cells of the gastrointestinal tract, including associated immune cells [3]. NOD2 is important for the proper functioning of the immune system, and any alteration of it can impair the body’s ability to kill bacteria and regulate immune responses. Under normal conditions, NOD2 increases the release of the cytokine’s interleukin-6 (IL-6) and interleukin-23 (IL-23) from dendritic cells and macrophages [3]. NOD2 also serves a role in downregulating the IL-23/interleukin-17 (IL-17) pathway [3]. Overall, NOD2 has key regulatory functions in autophagic processes, the IL-23 and Th17 inflammatory cascades, and gut homeostasis. The development of CD is exacerbated when the normal physiological functioning of NOD2 is altered [3]. It was also found that immune cells like dendritic cells, CD4-T cells, natural killer cells, and natural killer-T cells were especially active in CD patients with variations in NOD2 [3]. 

**Figure 2:**
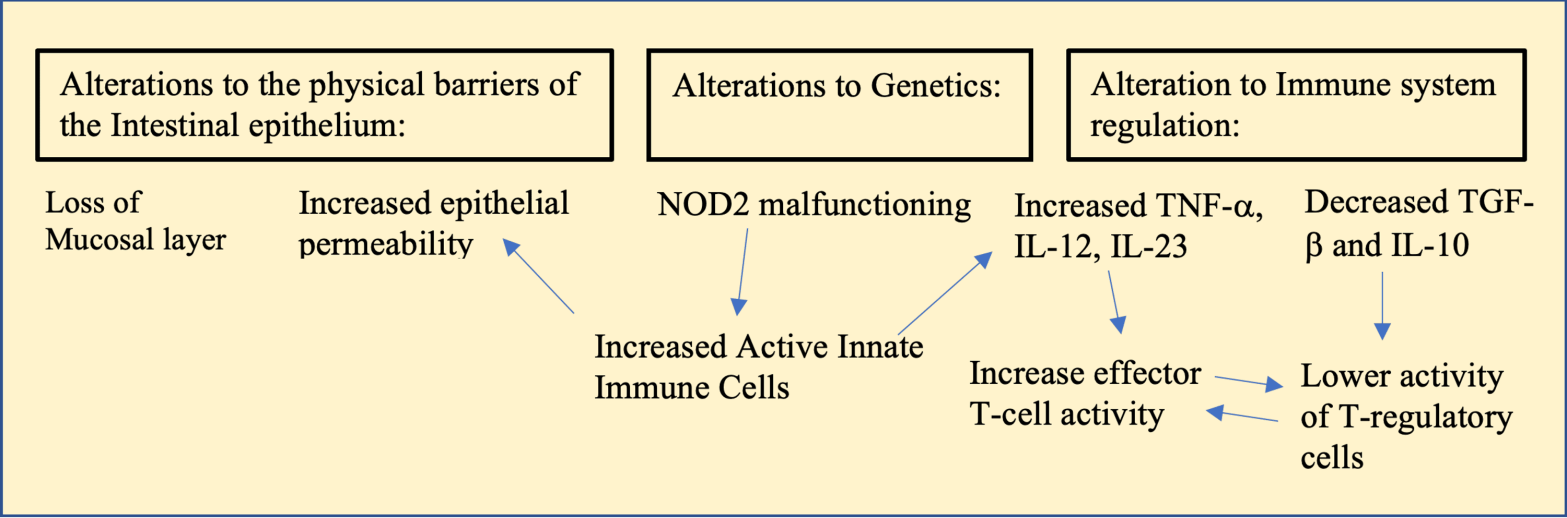
The inflammatory cascade of Crohn's disease Inflammatory cascades in inflammatory bowel disease (IBD) stem from the activation of tumor necrosis factor (TNF)-α, interleukins (IL), a dysfunctionally regulated nucleotide-binding oligomerization domain-containing protein 2 (NOD2), and downregulation of transforming growth factor (TGF)-β𝛽. Image credits: Haylie Smith

There are many dysregulated pathways associated with CD, as detailed in Figure 2. IL-23’s role in the IL-23/Th17 pathway is to induce and enhance CD4+ cells' production of IL-17 [4]. IL-17, in turn, plays a key role in priming T-cells and triggering the production of pro-inflammatory cytokines such as interleukin-1 (IL-1), IL-6, and tumor necrosis factor-α (TNF-α) [4]. Signal transducer and activator of transcription-3 (STAT3), Janus kinase-2 (JAK2), and tyrosine kinase-2 receptors are all key receptors involved in the IL-23/Th17 pathway [3]. Research has revealed that CD involves a Th1 cell-mediated immune response, wherein TNF-α is produced [5]. This release of TNF-α initiates a cascade of additional pro-inflammatory mediators, such as interferon-γ𝛾. Fascinatingly, despite TNF-α’s association with inflammatory processes, this biomarker also exhibited attributes of safeguarding the intestinal mucosa [5]. TNF-α contributes to the development of inflammation in the early stages of CD and is responsible for maintaining this inflammation in the later stages; however, it also plays a role in protecting the intestinal mucosa as well [5]. Anti-TNF-α modulators are commonly used to treat CD, but these medications can potentially inhibit TNF-α’s protection of intestinal mucosa rather than solely blocking the inflammation it causes [5]. CD is also associated with impairments in the regulatory pathways of the immune system. Interleukin-10 (IL-10) and T-regulatory cells have roles in limiting inflammation and maintaining proper homeostasis of the mucus [3]. However, this pathway can become defective in CD due to genetic factors and microbiota changes [3]. Studies have shown that certain intestinal microbiota species, such as *Clostridium*, help regulate this aspect of the immune system. However, these bacteria are less prominent in the microbiomes of patients with CD. In some cases of CD, there has been genetic variation in the IL-10 cytokines as well, which can impact the regulation of the immune system [3].  

There are theories that autophagy and endoplasmic reticulum (ER) stress, which is caused by misfolded proteins triggering the unfolded protein response (UPR), play a role in CD [3]. These two aspects of CD are closely associated with NOD2 [3]. This theory was further reinforced by a study that showed that variations in the genes responsible for protein folding and ER homeostasis, X-box binding protein 1 (XBP1) and anterior gradient protein 2 (AGR2), can be involved in CD and UC [6]. XBP1 helps maintain the homeostasis of the mucosa within the small bowels of the intestinal tract through its involvement with the UPR signaling pathway. Studies in mouse populations have shown that genetic deletions of XBP1 can induce ER stress, enhance epithelial cells’ sensitivity to immune system modulators, and alter Paneth cells in some way that triggers ileal inflammation [6]. Other studies in mouse populations have shown that genetic deletions of AGR2 result in epithelial ER stress, an impaired ability of Paneth cells to secrete protein, granulomatous inflammation, and an abnormal expansion of Paneth cells into the crypts and villi of the ileum [6].

Patient risk stratification, patient preference, clinical factors, age of onset, and comorbid complications are all assessed when choosing the optimal treatment strategy for those with CD [7]. The standard clinical treatment typically involves the use of steroids like prednisone to rapidly alleviate symptoms during the initiation of anti-TNF-α therapy [7]. Other agents available for CD that have therapeutic targets for modulating inflammation include mesalamine, azathioprine, and biologics such as anti-tumor necrosis factors [8]. Despite the various immunomodulatory therapies intended for CD treatment, patients continue to endure relapses and symptomatic flare-ups while using these medications [9]. Approximately 30-40% of individuals with CD either do not respond to TNF-α modulators or lose response to them over time [9]. The remission and flare-up rates of CD exemplify the necessity of new, additional therapies for CD. This review’s purpose is to investigate and evaluate some of these novel therapies that may prove beneficial in the treatment of CD resistant to TNF-α inhibitors.

## Review

There are several pharmaceuticals that have either recently been approved or are in development for the management of CD. A particularly promising aspect of advancements in CD treatment is the customization of medications to target specific stages of disease development, whether that be for inducing or maintaining clinical remission. All pharmaceuticals uncovered during our literature search are summarized in Table 1. Our review also reveals novel therapeutics that are being investigated for CD and are summarized in Table 2.

**Table 1:** Novel pharmaceuticals CD: Crohn's disease References: [10-12]

Drug Agent	Greatest Therapeutic Benefit	Pharmodynamics	Adverse Effects
Upadacitinib	More effective at maintaining remission rather than inducing it.	Orally administered Janus kinase inhibitor that suppresses interleukins and other proinflammatory mediators [10].	Gastrointestinal perforations may occur in patients that used non-steroidal anti-inflammatory drugs, nausea, elevated liver enzymes, and upper respiratory tract infections. The more serious side effects include malignancy and thrombosis [10].
Rizankizumab	Most effective at inducing and maintaining clinical remission among these novel pharmaceuticals.	Interleukin-23 antagonist that can only be administered via injection for CD [11].	Pneumonia, urinary tract infections, and COVID-19. It also causes disorders of the respiratory, integumentary, and musculoskeletal systems [11].Myocardial infarctions, cerebrovascular accidents, and pruritus were noted as common adverse events [11].
Vedolizumab	Induction and maintenance of clinical remission with added benefits of avoiding systemic immunosuppression.	Administered intravenously to block immune cell migration to the gastrointestinal tract via α4β7 integrin antagonism [12].	Nasopharyngitis, hypersensitivity reactions, and upper respiratory infections [12].

**Table 2:** Novel therapies FMT: Fecal microbiota transplantation, MSCs: Mesenchymal stem cells, HSCs: Hematopoietic stem cells, CD: Crohn's disease References: [8, 13-19].

Therapy	Greatest Therapeutic Benefit	Mechanism of Action	Important Findings	Adverse Effects
Synbiotics (Synergy 1)	Improving clinical symptoms in patients with active CD who want to avoid the adverse effects of other treatment modalities [13].	Rectifying gut dysbiosis in hopes of reducing inflammation in an individual with a dysregulated immune system through an increase in Bifidobacterial species harboring the intestines [13].	Significant improvement in clinical outcomes, reduced histopathological activity, and increased proportions of Bifidobacterial species with Synergy 1 [13].	Systemic infections, adverse metabolic activity, potential overstimulation of the immune system in susceptible individuals, and gastrointestinal side effects [8].
FMT	87.5% of patients undergoing single-dose FMT showed higher rates of steroid free clinical remission when compared to 44.4% of patients in a placebo group [14].	Rectifying gut dysbiosis by transplanting fecal microbiota from healthy donors into the gastrointestinal tract of those with gut dysbiosis [14].	Administration of FMT through capsule delivery is associated with higher rates of adverse effects than colonoscopy guided FMT [15].	Abdominal pain, diarrhea, *Clostridium difficile* infections [15].
Stem Cell Therapy	MSCs can help resolve fistulas by secreting growth factors to promote wound healing [16].	Intravenously administered MSCs can travel to and colonize the intestinal injury, ultimately assisting with wound healing, controlling inflammation, and improving microcirculation [16]. HSCs allow the body to produce new lymphoid and myeloid cells to help correct an abnormal immune response [16].	91% of CD patients given HSCs reached clinical remission for the first year, 57% for the third year, and 19% for the fifth year following treatment [16,17]. No significant difference in long term clinical remission rates between HSC therapy and placebo [16].	No serious adverse effects with MSCs other than fever and peri-procedural proctalgia and abscesses [18]. HSCs associated with viral infections and graft rejection [16,19].

Novel pharmaceuticals

**Figure 3:**
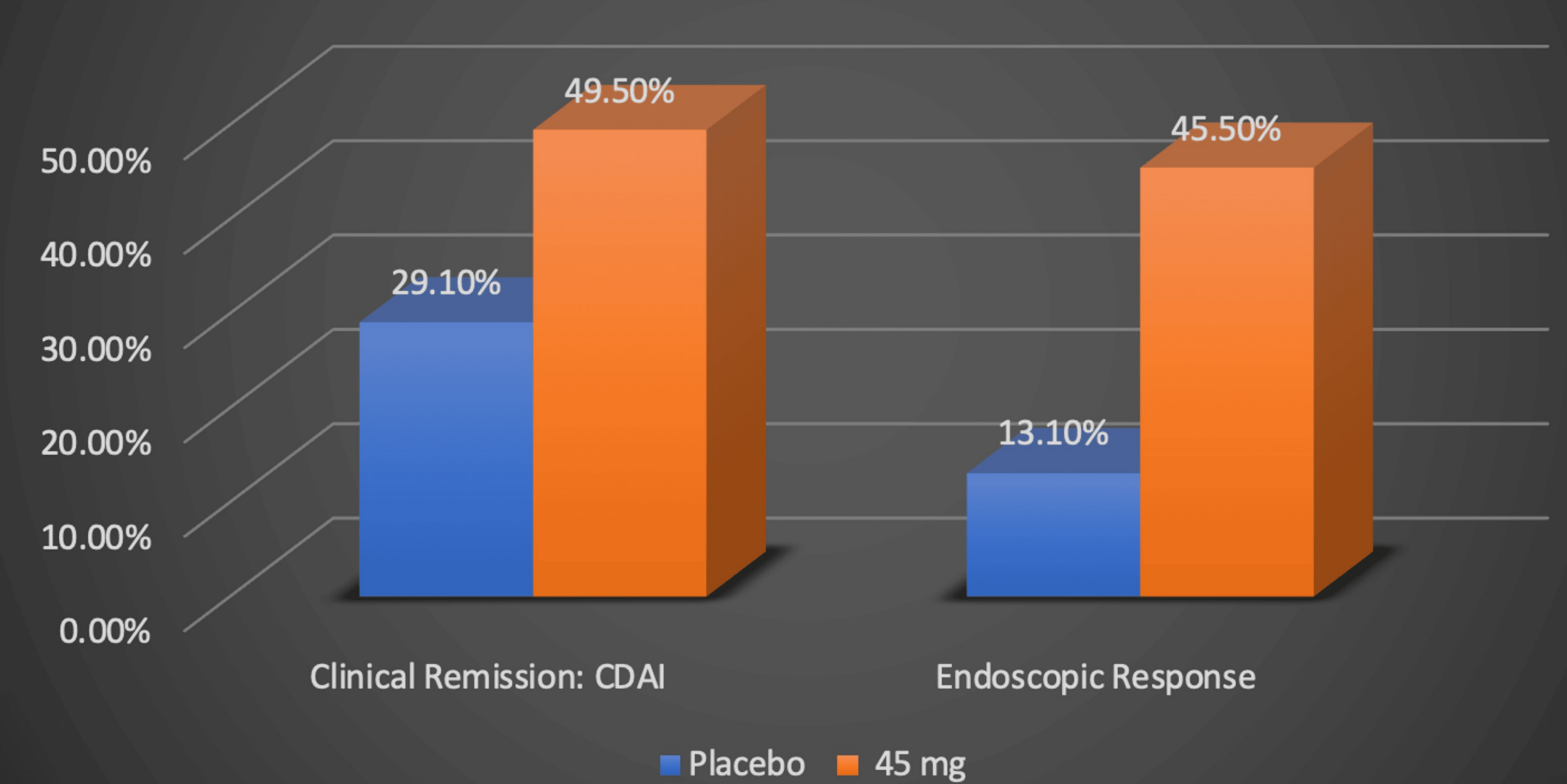
Upadacitinib for the induction of clinical remission Numerical data was taken from the U-EXCEL clinical trial [20]. Percentages represent the proportion of Crohn's disease patients successfully responding to treatment. CDAI: Crohn's disease activity index (CDAI), mg: milligram Image credits: Blake Smith

Upadicitinib is a drug that is known to block the JAK-1 enzyme, thereby inhibiting growth factor and cytokine signaling in a pathway commonly associated with immune-mediated inflammatory disease [10]. It is already approved by the U.S. Food and Drug Administration (FDA) for rheumatoid arthritis, psoriatic arthritis, and atopic dermatitis; however, it is still undergoing clinical trials for its use in UC and CD [10]. Phase three clinical trials, U-EXCEL and U-EXCEED, were conducted involving individuals with moderate-to-severe CD who were divided into two groups: the placebo group and those receiving upadacitinib [20]. Those who were a part of the upadacitinib group received 45 milligrams (mg) of the medication every day for 12 weeks during the induction phase. The subjects who responded to the induction therapy were then assigned to either take 15 mg, 30 mg, or a placebo daily for 52 weeks as part of the maintenance phase in the U-ENDURE clinical trial [20]. For the induction testing, they found that 49.5% of those taking the 45 mg of upadicitinib reached clinical remission compared to 29.1% of those in the placebo group, as illustrated by the original graph in Figure 3 [20].

**Figure 4:**
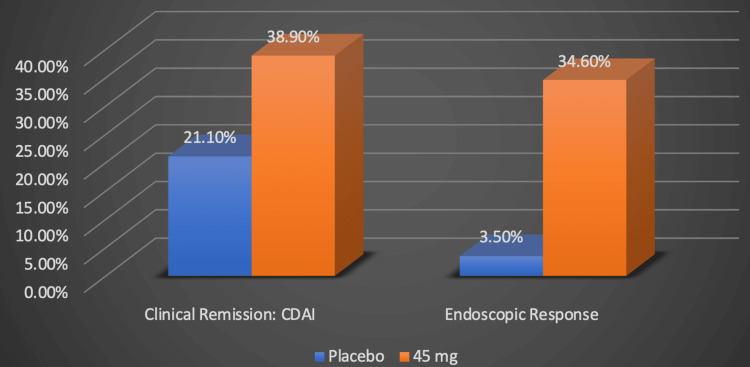
Upadacitinib's efficacy in inducing clinical remission Numerical data was taken from the U-EXCEED clinical trial [20]. Percentages represent the proportion of Crohn's disease patients successfully responding to treatment. CDAI: Crohn's disease activity index, mg: milligram Image credits: Blake Smith

Another trial, U-EXCEED, also investigated upatacitinib’s efficacy in inducing clinical remission, as illustrated in the original graph in Figure 4 [20].

**Figure 5:**
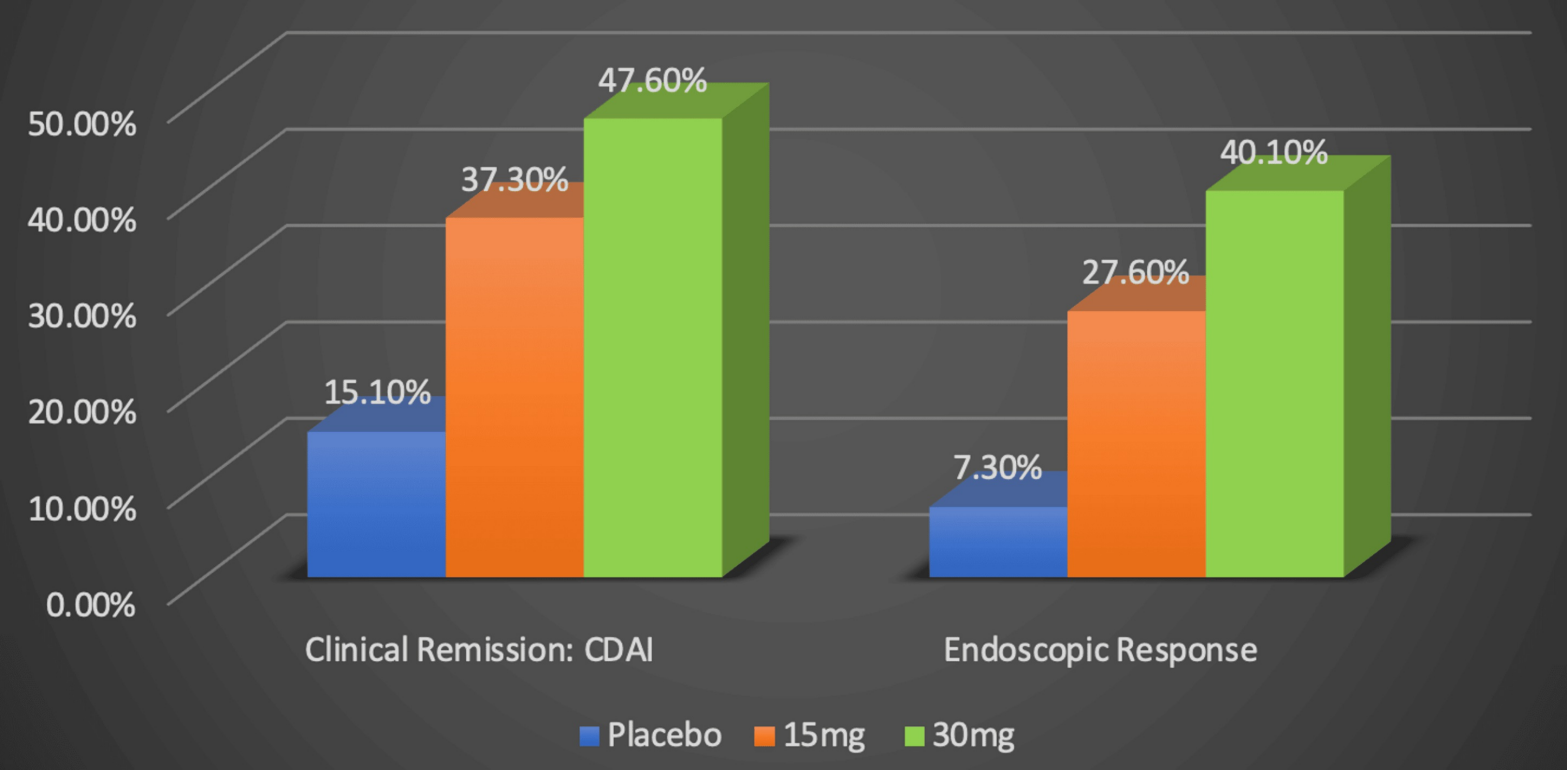
Upadacitinib's efficacy in maintaining remission Numerical data was taken from the U-ENDURE trial [20]. Percentages represent the proportion of Crohn's disease patients successfully responding to treatment. CDAI: Crohn's disease activity index, mg: milligram. Image credits: Blake Smith

In the U-ENDURE trial regarding upadacitinib’s ability to maintain clinical remission, 37.3% of patients given 15 mg and 47.6% of those given 30 mg reached clinical remission versus 15.1% of patients given placebo, as illustrated by the original graph in Figure 5 [20]. They also found that 27.6% of those given 15 mg and 40.1% of those given 30 mg of the medication had an endoscopic response, which was classified as reducing their baseline simple endoscopic score for CD by 50%, compared to 7.3% of those on the placebo [20].

Anemia, neutropenia, creatine kinase elevations, hepatic issues, and infections that are severe or opportunistic are reported adverse effects of upadacitinib [20]. They also found that those who were given higher doses of the medication were more prone to developing herpes zoster infections [20]. The study found that a small number of those who took 45 mg of upadacitinib developed perforations more often than the other groups in the studies, but those individuals were found to have an active flare-up or complications of CD like deep ulcers and strictures. The study concluded that it cannot be determined if these perforations were caused by upadacitinib or not [20]. Other studies have shown that gastrointestinal perforations may occur in patients who use non-steroidal anti-inflammatory drugs with upadacitinib [10]. Other adverse effects included nausea, elevated liver enzymes, and upper respiratory tract infections. The more serious side effects include malignancy and thrombosis [10]. It is recommended that this medication not be administered to individuals on biological disease-modifying antirheumatic drugs (DMARDs), on immunosuppressants, with absolute lymphocyte counts below 500 cells/mm^3, with absolute neutrophil counts below 1000 cells/mm^3^, with liver impairments, or who received live vaccinations before or during therapy [10]. It is also recommended not to take this medication while pregnant or breastfeeding due to its potential teratogenic effects. It was advised that patients should be assessed before and during therapy for things like tuberculosis, an abnormal complete blood count (CBC), and liver functionality [10]. The cytochrome P450 system metabolizes upadacitinib, with its terminal half-life ranging from 6 to 16 hours and its functional half-life at a steady state ranging from three to four hours [10].

**Figure 6:**
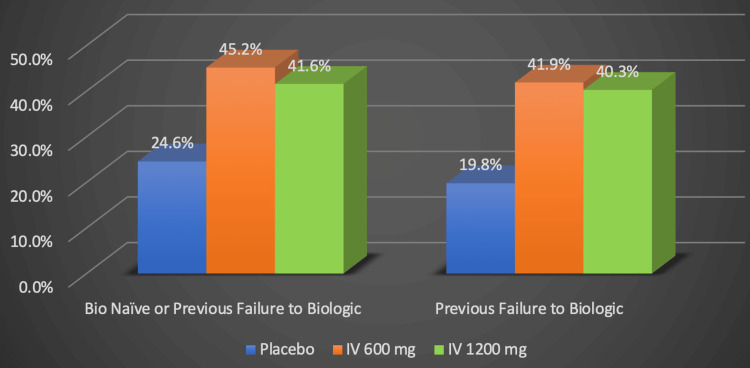
Risankizumab's efficacy in inducing clinical remission: Crohn's Disease Activity Index Numerical data was taken from the ADVANCE and MOTIVATE clinical trials [22,23]. Percentages represent the proportion of Crohn's disease patients successfully responding to treatment. IV: Intravenous (IV), mg: milligram Image credits: Blake Smith

Risankizumab is another medication that operates in a similar manner. Risankizumab is an injectable antibody that targets IL-23 and is indicated for moderate-to-severe CD [21]. The ADVANCE and MOTIVATE clinical trials followed patients for 12 weeks to test risankizumab’s ability to induce clinical remission using 600 mg and 1200 mg of the drug [22,23]. They found that there was no benefit in using 1200 mg versus 600 mg for induction [22,23]. Figure 6 illustrates that 45% of the patients in ADVANCE, which was a clinical trial with a sample population consisting of individuals who had either never used biologics or had previous biological failures, reached clinical remission by week 12 using 600 mg of risankizumab, compared to the 25.2% of individuals in the placebo group [22,23]. In the MOTIVATE study, which exclusively involved patients who had previously failed to respond to biologics, 42% of patients treated with this medication at 600 mg achieved clinical remission by week 12, compared to 19.8% of patients treated with a placebo [22,23]. 

**Figure 7:**
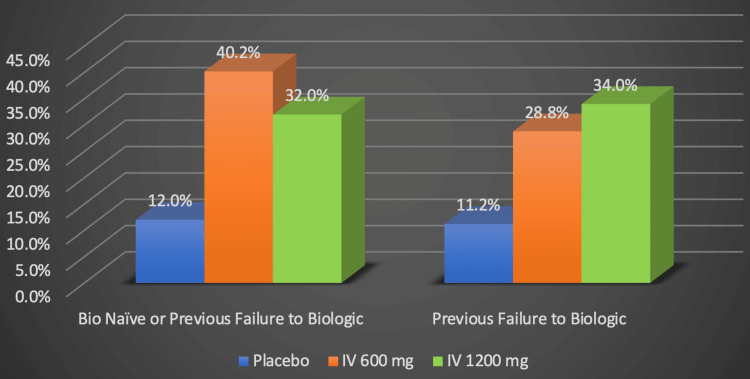
Risankizumab's efficacy in inducing remission: endoscopic response Numerical data was taken from the ADVANCE and MOTIVATE clinical trials [22,23]. Percentages represent the proportion of Crohn's disease patients successfully responding to treatment. IV: Intravenous (IV), mg: milligram Image credits: Blake Smith

As illustrated by the original graph in Figure 7, 40% of participants in the ADVANCE trial and 29% of participants in the MOTIVATE trial had endoscopic responses [22,23]. Endoscopic response was defined as having a simple endoscopic score under four and having decreased by at least two points [22,23]. In contrast, only 12% and 11% of individuals in the placebo groups of the respective trials achieved similar results [22,23]. The ADVANCE clinical trial also concluded that those who had not received biologics before responded better to the medication than those who did [22,23].

**Figure 8:**
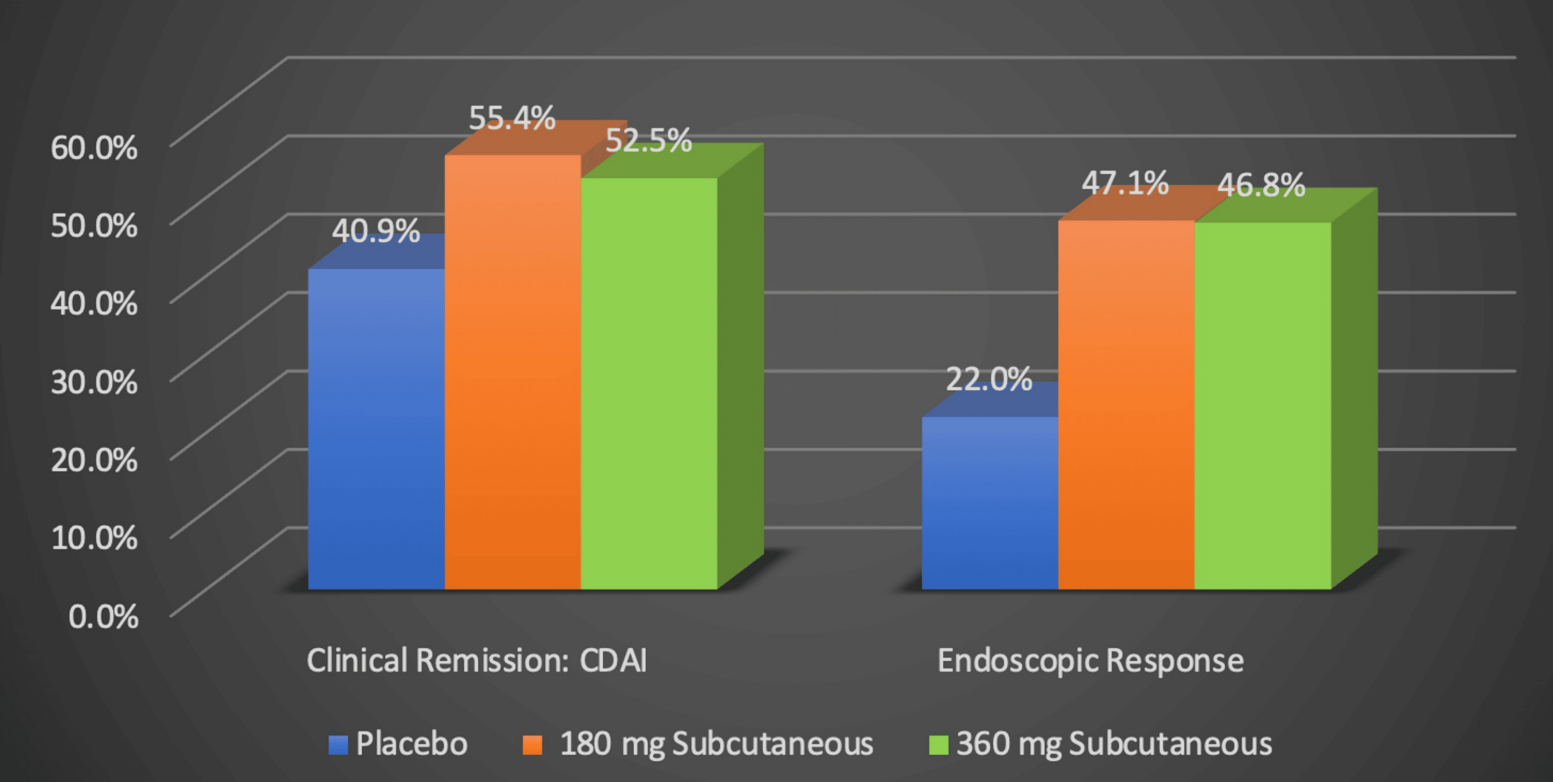
Risankizumab's efficacy in maintaining remission Numerical data was taken from the FORTIFY clinical trial [21,22]. Percentages represent the proportion of Crohn's disease patients successfully responding to treatment. CDAI: Crohn's disease activity index, mg: milligram Image Credits: Blake Smith

The FORTIFY trial, represented by the original graph in Figure 8, targeted individuals who reached clinical remission with this medication during the induction phase [21,22]. It aimed to assess risankizumab as a potential therapy for maintaining clinical remission [21,22]. Maintenance therapy with either 180 mg or 360 mg of risankizumab demonstrated a significantly greater response to achieving endoscopic and clinical remission when compared to a placebo [21,22]. The subjects were given a dose of their assigned treatment every eight weeks after their 12-week induction period and were monitored for 52 weeks in total in this study [21,22].

The pharmacokinetics of risankizumab follow closely what fellow immunoglobulin G-1 antibodies do [24]. Risankizumab had similar pharmacokinetics in patients with CD and psoriasis; the only factors that impacted risankizumab’s pharmacokinetics were weight and albumin levels [24]. Reduced weight is associated with lower albumin levels, which leads to lower steady-state volumes of distribution, terminal phase half-lives, and clearance rates of the medication [24]. Risankizumab is characterized as having linear pharmacokinetics and a terminal half-life of 28 days [25]. It is metabolized into amino acids for elimination and does not require hepatic or renal systems for this process [25]. Previous studies show that adverse effects of risankizumab are more common within the first three months of starting, with the risk decreasing over time [11]. Some common adverse effects in general include infections like pneumonia, urinary tract infections, and COVID-19 [11]. It also causes disorders of the respiratory, integumentary, and musculoskeletal systems [11]. Myocardial infarctions, cerebrovascular accidents, and pruritus were noted as common adverse events associated with risankizumab’s use, in contrast to adverse effects like headache, nausea, diarrhea, abdominal pain, and nasopharyngitis that were noted in previous studies [11].  

**Figure 9:**
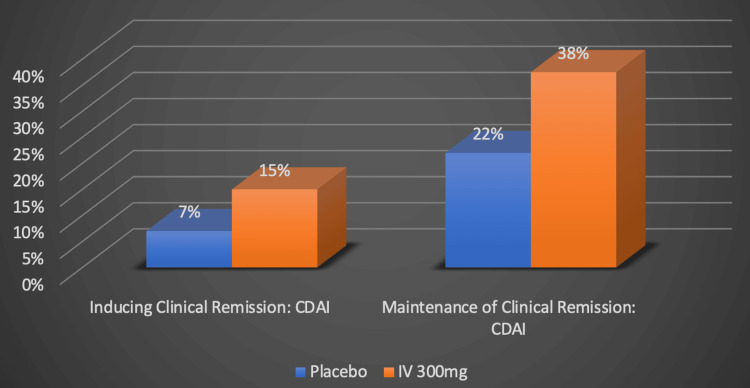
Vedolizumab's efficacy in inducing and maintaining remission Numerical data was taken from the GEMINI-II clinical trial [27]. Percentages represent the proportion of Crohn's disease patients successfully responding to treatment. CDAI: Crohn's disease activity index, IV: Intravenous, mg: milligram. Image credits: Blake Smith

Vedolizumab is another immunomodulator being investigated for the treatment of CD that acts through a different mechanism of action. Vedolizumab is an anti-α 4β 7 integrin that helps prevent inflammation by selectively blocking lymphocyte tracking and migration to the gut [12]. A therapeutic advantage of vedolizumab is that it prevents local inflammation specifically confined to the gastrointestinal tract, as opposed to overall systemic immunosuppression [12]. It accomplishes this by impeding α 4β 7 integrin’s interaction with mucosal addressing cell adhesion molecule-1, ultimately preventing lymphocyte translocation from the blood into the gut lumen [12]. Three hundred mg of vedolizumab is administered intravenously to those with severe, active UC and CD, but it is unique in that it can also be given subcutaneously. Its terminal half-life is estimated to be 25.5 days and it is metabolized into peptides and amino acids that are later excreted by the kidneys [26]. Research has validated the advantages of vedolizumab as a treatment for CD. It was reported that 15% of patients receiving vedolizumab achieved clinical remission, defined as having a CDAI of less than 150 [27]. This is in contrast to the 7% of patients who had achieved clinical remission in the placebo group, as depicted by the original graph in Figure 9 [27].

Substantial differences in clinical remission rates were seen in anti-TNF-α resistant individuals who were later treated with vedolizumab. When compared to a placebo at a 52-week follow-up, vedolizumab exhibited a maintenance remission rate of 46.4% when given subcutaneously, as opposed to 28.8% in the placebo group [12]. Remarkably, even among CD patients who had previously shown resistance to TNF-α inhibitor therapy and did not receive corticosteroids, the remission rates remained impressive at 46.2% [12]. Vedolizumab has shown promising results in its efficacy for anti-TNF-α resistant cases but has drawbacks. Nasopharyngitis (9.1%), hypersensitivity reactions (8.7%), and upper respiratory infections (6.2%) were adverse events reported by those in the vedolizumab treatment group [12]. Vedolizumab has demonstrated its value as a second-line treatment option for those with CD resistant to anti-TNF-α therapies. Due to its favorable safety profile, vedolizumab should be considered as a first-line treatment for steroid-dependent and steroid-refractory CD patients who are not responding to or are ineligible for immunosuppressant agents [27]. Vedolizumab may be the superior treatment option for CD patients who need to avoid systemic immunosuppression, such as the elderly and those at risk of opportunistic infections [27]. 

Novel therapies

The compositional makeup of the gut microbiome is pivotal in maintaining gut homeostasis and regulating inflammation. Fortunately, ongoing research and development has recognized this and is investigating the gut microbiome as a potential therapeutic target for CD. Recent studies have highlighted the innate immune system’s role in instigating imbalances between the beneficial microbes and commensal microflora that reside in the gastrointestinal tract [8]. Increased gut composition of the commensal microbes *Lactobacillus* and *Bifidobacterium* may be a valuable resource in IBD prevention, while higher levels of *Escherichia coli* and *Bacteroidetes* are associated with CD [2]. This finding led to researchers’ turning towards prebiotics, probiotics, and synbiotics as alternative therapies for CD [13]. Multiple mechanisms of action have been reported as a way to explain probiotics’ benefits in CD management, such as suppressing pathogenic bacterial overgrowth through the release of antimicrobial byproducts, triggering or regulating immune response, enhancing epithelial barrier activity, and suppressing the proliferation of T-cells [8]. Probiotics are capable of suppressing pathogenic enteric bacteria by blocking their binding to the epithelial layer, decreasing luminal pH in hopes of creating an inhospitable environment, secreting bactericidal proteins, and preventing colonization [8]. Probiotics improve the functioning of the epithelial and mucosal barrier by increasing short-chain fatty acid production, mucus secretion, and the integrity of the epithelial and mucosal barrier [8]. Some probiotics can alter the immune system’s modulatory processes through the upregulation of IL-10, transforming growth factor-β𝛽, and immunoglobulin-A production while simultaneously decreasing levels of the pro-inflammatory mediator, TNF-α [8]. Adverse side effects such as systemic infections, gene transfer, gastrointestinal side effects, and excessive immune stimulation have only been theorized to accompany the use of synbiotics based on the results from various case reports, clinical trials, and experimental models [28]. The most common synbiotic formulas consist of *Lactobacillus* and/or *Bifidobacteria* bacterial strains along with *fructooligosaccharides* and/or inulin to further promote their growth [13].  Synergy-1 is an example of one of these synbiotics being investigated as a potential therapy for the management of CD. Significant improvement in clinical outcomes reduced histopathological activity, and an increase in *Bifidobacteria* harboring the intestines were all reported in patients taking Synergy-1 when compared to a placebo [13]. Biopsies of mucosal specimens also showed a significant decrease in TNF-α after three months; however, this finding did not persist after six months [13]. Synbiotic 2000 is a different synbiotic formula consisting of four probiotics (*Pediacoccus pentosecens*, *Lactobacillus affinolactis*, *Lactobacillus paracasei susp paracasei 19*, and *Lactobacillus plantarum 2362*) and four prebiotics (β-glucan, inulin, pectin, and resistant starch) [13]. Unfortunately, when Synbiotic 2000 was compared to a placebo in CD patients who previously underwent surgical resection, no significant improvements in clinical, laboratory, or endoscopic activity were reported [13]. This novel area of research on synbiotics as a potential therapy for CD includes studies with significant limitations, such as small study populations in combination with a lack of analysis of concomitant treatments. These limitations may explain the contradictory findings found on synbiotics and their use in those with CD. Additional research investigating the efficacy of prebiotics, probiotics, and synbiotics in CD management is needed for confirmatory results.  

Fecal microbiota transplantation (FMT) is another therapy with the intention of rectifying the abnormal compositional makeup of the gut microbiome found in patients suffering from CD. FMT is accomplished by transplanting fecal microbiota from healthy donors into the gastrointestinal tract of those with intestinal dysbiosis [14]. FMT is already a successful treatment therapy for those with *Clostridium difficile* infections and is now being investigated as an alternative therapy for CD. FMT can be administered directly to the colon via colonoscopy or enema, or from the upper gastrointestinal tract through the ingestion of a capsule [15]. In a randomized clinical trial, 87.5% of patients undergoing a single-dose FMT showed a higher rate of steroid-free clinical remission in contrast to the 44.4% of patients in the placebo group [14]. Thirty-nine percent of individuals undergoing FMT reported side effects of abdominal pain and diarrhea [15]. A more serious complication of FMT is *Clostridium difficile* infection, which was reported by 5.3% of the study population undergoing FMT [15]. The administration of FMT through capsule delivery is associated with higher rates of adverse effects [15]. Preliminary studies have suggested FMT’s potential in alleviating CD; however, these studies included small sample sizes. Large control studies are still needed for confirmatory results. FMT may be a promising therapy for CD in the near future due to its favorable safety profile compared to current CD treatments readily available today.  

Stem cell transplantation is a new technique that is being researched as a potential treatment for IBD. There are various types of stem cells being researched for IBD. Hematopoietic stem cells (HSCs) and mesenchymal stem cells (MSCs) are the two cell lines that research has focused on the most for the treatment of IBD [16]. MSCs are administered intravenously for general systemic treatment of CD or can be directly injected into fistulas that develop because of the disease [18]. However, rectovaginal fistulas do not respond well to this treatment option [18]. The mechanism of action of MSCs, particularly the bone marrow-mesenchymal stem cells (BM-MSC), is to travel to the intestinal injury and colonize it when administered intravenously [16]. When they colonize the intestines, the stem cells can help with controlling inflammation, wound healing, and improving local microcirculation. A few studies have reported that MSCs' mechanism of action can help with reducing inflammation and symptoms of CD in general [16]. Most of the research done so far on MSCs shows that they can help resolve fistulas by secreting pro-wound healing growth factors, but they do not appear to help prevent recurrences of these fistulas [16]. More research needs to be done for this type of stem cell use in CD. The safety of stem cell transplants so far has shown that MSCs do not typically cause any serious adverse effects [19]. Some studies found that MSCs can cause fever, suppress tumors, and inhibit tumorigenesis [16].

As of today, most of the research is around HSCs, as they are easier to access for research since they can be obtained from peripheral blood, bone marrow, or cord blood [16]. These stem cells can improve the symptoms of patients with UC and CD by allowing the body to produce new immune cells and by reducing immune-mediated inflammation [16]. Various studies have been done on HSCs being used for CD [16]. Ninety-one percent of CD patients treated with HSC transplantation remained in clinical remission at the one-year follow-up, 57% remained in clinical remission at the three-year follow-up, and 19% were still in clinical remission at the five-year follow-up [16,17]. Other research has found that transplanting HSCs with cyclophosphamide and granulocyte-stimulating factors can improve patient outcomes, but a majority of those who received this treatment regimen were experiencing symptoms again at the three-year follow-up [16,29]. Given the risk of relapse and the smaller population sizes included in trials so far, more research needs to be done before establishing stem cell therapy as a viable treatment for CD [16]. HSCs tend to have an increased risk of adverse effects like viral infections since the patient’s immunity is reduced during the mobilization of the HSCs [19]. HSCs’ adverse effects depend on whether they are autologous or allogeneic [16]. If it's autologous, the genetic predisposition to CD is still there, but it resets the immune system and can lead to long-term remission if there are no triggers for CD. Allogeneic transplantation always has the risk of graft rejection [16]. 

Discussion

**Figure 10:**
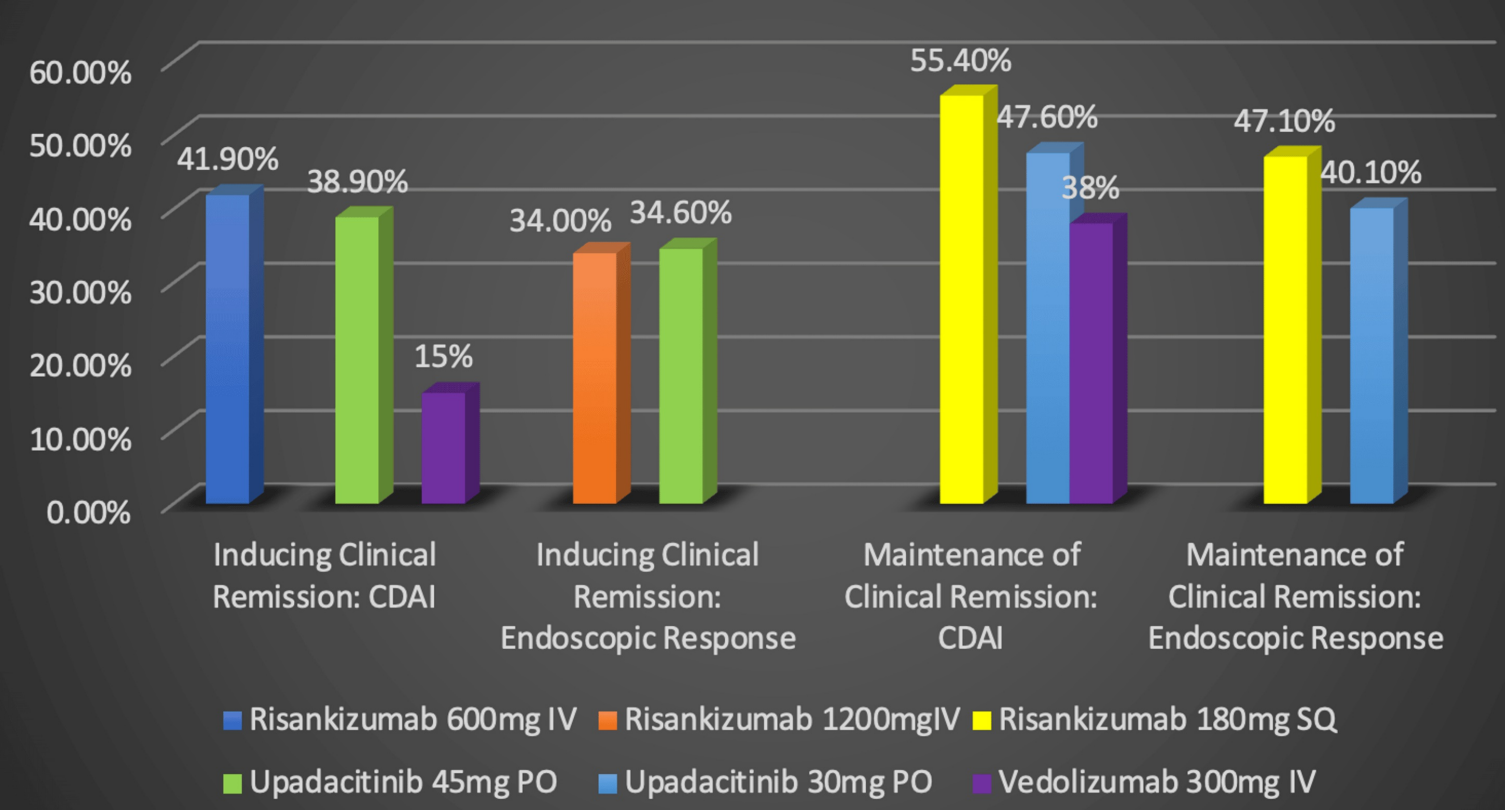
Comparison of novel pharmaceuticals Data taken from the MOTIVATE, FORTIFY, U-EXCEED, U-ENDURE, and GEMINI-II clinical trials [20-23,27]. Percentages represent the proportion of Crohn's disease patients successfully responding to treatment. CDAI: Crohn's disease activity index, IV: Intravenous, SQ: Subcutaneous, PO: Per os, mg: Milligram Image Credits: Blake Smith

Future clinicians may be able to optimize CD treatment by deliberately selecting the right pharmaceutical at the most appropriate stage of the patient’s disease progression. This narrative review analyzed and compared data from drug trials involving risankizumab, upadacitinib, and vedolizumab. This review's comparison focused on trials with similar study durations and populations. Efficacy was assessed using the CDAI and endoscopic evaluation. Factors such as dosage, route of administration, and pharmaceutical selection were considered. This review compared these three drugs in terms of their capability of inducing and maintaining remission, as depicted in Figure 10. However, the data is not confirmatory due to a lack of studies that directly compare these novel pharmaceuticals against one another. 

When comparing novel pharmaceuticals, we deliberately researched pharmaceuticals that had recently completed clinical trials or had limited research on them. This study analyzed data from studies that had the most resemblance to one another to the best of our ability. Data on risankizumab was extracted from the MOTIVATE and FORTIFY drug trials. This narrative review’s pharmaceutical comparison incorporated data on upadacitnib from the U-EXCEED and U-ENDURE drug trials. Data on vedolizumab was included in this narrative review’s analysis of the GEMINI-II drug trial. 

We chose to analyze data on risankizumab from the MOTIVATE and FORTIFY trials due to their similarities with studies investigating upadacitinib and vedolizumab. The MOTIVATE drug trial consisted of 569 patients who had previously failed biologic treatment [22,23]. The FORTIFY trial included 462 patients, with 73% experiencing previous biologic failure [21,22]. The trials regarding risankizumab that were included in the comparison also shared the same measurement outcomes as the studies investigating upadacitinib and vedolizumab (CDAI and endoscopic response). The induction trial on risankisumab also aligns with the trial duration of the study on upadacitinib, lasting 12 weeks. However, there is some disparity between the trials on vedolizumab and risankizumab due to the trial duration of vedolizumab being limited to six weeks. Risankizumab’s FORTIFY maintenance trial is similar in time length to the vedolizumab and upadacitnib maintenance trials at 52 weeks [22]. 

Data on upadacitnib was extracted from the U-EXCEED and U-ENDURE trials [20]. These trials were favorable to incorporate into our comparison due to their similarities with the risankizumab and vedolizumab trials. The patient population in the upadacitinib induction trial consisted of 495 patients, all of whom had previously failed biologic treatments [20]. The U-ENDURE trial had 502 participants, with 75.6% of participants in the 30 mg treatment group previously failing biologic treatment [20]. Both the induction and maintenance trials on upadacitnib measured the efficacy of upadacitnib via the Crohn's Disease Activity Index (CDAI) and endoscopic evaluation. The trial duration of the induction and maintenance studies of upadacitninb lasted 12 and 52 weeks, respectively [20]. 

Vedolizumab’s data was extracted from the GEMINI-II trials [27]. Potential drug trials investigating vedolizumab were limited, but the trials included in this narrative review’s comparison were the most similar to trials investigating risankizumab and upadacitinib. 368 of the participants in the GEMINI-II induction trials and 461 participants in the GEMINI-II maintenance drug trials, respectively, had either no response or adverse effects when previously using anti-TNF-α, steroid, or immunosuppressive medications [27]. Vedolizumab’s efficacy was assessed only using CDAI. The drug trial on vedolizumab only lasted for six weeks in the induction study; however, the maintenance study assessing vedolizumab’s effectiveness lasted for the same amount of time as the risankizumab and upadacitinib studies, 52 weeks [27].

Risankizumab appeared to be the superior treatment for both inducing and maintaining clinical remission in patients resistant to or contraindicated to biologics. Its greatest strength seemed to be its ability to maintain clinical remission. Risankizumab appeared to be the superior treatment for managing patients currently in clinical remission when administered subcutaneously at a dose of 180 mg [21-23]. It was also proven effective at inducing clinical remission based on the CDAI measurement when intravenously administered at a dose of 600 mg and at 1200 mg if assessed based on endoscopic response [11,23]. Risankizumab should be avoided in those susceptible to pneumonia, urinary tract infections, and COVID-19 [11].

Upadacitinib’s greatest strength seemed to be its ability to maintain clinical remission in those who previously failed treatment with biologics when administered at a daily dose of 30 mg [20]. Its ability to do so was found to be to a lesser extent than Risankizumab's ability. Upadacitinib also showed promising results for inducing clinical remission when administered at a daily oral dose of 45 mg, but again, to less of an extent than risankizumab. Despite upadacitinib’s promising results for those resistant to TNF-α inhibitors, it has adverse effects and may want to be avoided in those susceptible to anemia, neutropenia, elevated creatine kinase levels, hepatic issues, malignancies, thrombus development, and serious or opportunistic infections [10,20]. It was recommended that this drug be avoided in individuals with previous hypersensitivity reactions, on biological DMARDS, on immunosuppressants with absolute lymphocyte counts below 500 cells/mm^3^ or absolute neutrophil counts below 1000 cells/mm^3^, with liver impairments, or who received live vaccines before or during therapy [10]. Pregnant or breastfeeding women should not be administered this medication due to its potential teratogenic effects [10].

Vedolizumab appeared to be the least effective among these novel pharmaceuticals for both inducing and maintaining clinical remission based on CDAI; however, more studies are needed for confirmation. Only 15% of patients treated intravenously with 300 mg of vedolizumab showed CDAI scores of less than 150 after six weeks of treatment [30]. At the 52-week follow-up, 38% of patients who received the same dose intravenously maintained clinical remission based on CDAI [30]. The data analyzed on vedolizumab came from studies that did not include endoscopic response assessment as a measure of its efficacy. Additionally, the study evaluating vedolizumab’s ability to initiate remission was constrained by a six-week timeframe, in contrast to the 12-week durations of the upadacitinib and risankizumab studies. This discrepancy could potentially affect our assessment when comparing vedolizumab's effectiveness with that of upadacitinib and risankizumab. Vedolizumab could represent a preferable treatment choice for patients with CD who require the avoidance of systemic immunosuppression, particularly among elderly individuals and those susceptible to opportunistic infections [27].

The research on stem cells as a potential treatment option for CD is still in its preliminary stages and has shown some conflicting data on its efficacy in treating CD. More research needs to be done on this treatment option before it becomes established as a viable treatment option for CD. This is also true for FMT and synbiotics as well. All of these treatments are being researched with small sample sizes. More research needs to be done to confirm the safety and efficacy of these treatments.

## Conclusions

The latest advancements in the management of CD have shown encouraging results, especially for individuals who have exhausted other treatment options due to their resistance or intolerance to TNF-$\alpha$ inhibitors. Alternative pharmaceuticals that diverge from the conventional standard of care consist of upadacitinib, risankizumab, and vedolizumab. Some of these novel drugs not only offer a potential solution for CD patients facing resistance to TNF-$\alpha$ inhibitors but may also provide a superior alternative for individuals prone to opportunistic infections or the adverse effects associated with existing treatments. Synergy-1 synbiotics, fecal microbiota transplantation (FMT), and stem cell therapy are emerging treatment options that have so far shown conflicting results, but they seem to have some promising aspects for the treatment of CD.
